# Effects of increasing levels of lasalocid supplementation on growth performance, serum biochemistry, ruminal fermentation profile, *in vitro* nutrient digestibility, and gas production of growing goats

**DOI:** 10.3389/fvets.2023.1181426

**Published:** 2023-06-12

**Authors:** Saeid M. Basmaeil, Gamaleldin M. Suliman, Maged A. Al Garadi, Mohammed A. Al-Badwi, Mutassim M. Abdelrahman, Fahad S. Al-Harbi, Ahmed M. El-Waziry, Ibrahim A. Alhidary, Ayman A. Swelum

**Affiliations:** ^1^Department of Animal Production, College of Food and Agriculture Science, King Saud University, Riyadh, Saudi Arabia; ^2^Department of Animal and Fish Production, Faculty of Agriculture, Alexandria University, Alexandria, Egypt

**Keywords:** Animal Welfare, feed additive, gas production, performance, ionophore

## Abstract

**Introduction:**

Lasalocid is a feed additive widely used in ruminant nutrition and plays a crucial role in improving livestock productivity, digestibility, immunity, and overall wellbeing. The current study was conducted to investigate the effect of different levels of lasalocid (LAS) supplementation on growth performance, serum biochemistry, ruminal fermentation profile, *in vitro* nutrient digestibility, and gas production of growing goats.

**Methods:**

A total of 60 growing Aardi male goats with an average body weight of ~17.12 kg (3-month-old) were used for an 84-day trial. Animals were randomly divided into four treatment groups with 5 replicates of 3 goats each. All four groups were provided with a basal diet supplemented with lasalocid (LAS) at 0 (without supplementation; LAS0), 10 (LAS10), 20 (LAS20), or 30 (LAS30) ppm LAS/kg dry matter (DM). Feed intake was measured weekly, and goats were weighed every 2 weeks for an evaluation of the performance parameters. Blood samples were collected for the measurement of biochemical variables. *In vitro* nutrient digestibility and gas production were evaluated.

**Results and discussion:**

The supplementation of LAS at level 30 ppm/kg DM increased (*P* < 0.05) the body weight gain and average daily gain without linear or quadratic effect. The serum concentrations of high-density lipoprotein were significantly (*P* < 0.05) higher in the LAS20 group than in other groups with linear and quadratic effects, while low-density lipoprotein concentration was significantly lower in the LAS20 group than in LAS0 and LAS30 with a linear effect. Different levels of lasalocid supplementation had no effect on the ruminal fermentation profile, *in vitro* gas production, and nutrient digestibility. In conclusion, the addition of LAS (20–30 ppm/kg DM) to the goat's diet can improve the growth performance and lipoprotein profile.

## Introduction

Goats are essential livestock in tropical and subtropical countries and consider a good source of milk and meat production. In general, low-quality forage leads to high acetate and low propionate production during ruminal fermentation (1, 2). Alternatively, concentrated feed, with a high rumen degradable protein content, often characterizes by an imbalance between ruminal protein and carbohydrate fermentation, which can lead to excess production and accumulation of gas especially ammonia in the rumen (3, 4); therefore, impaction, ruminal acidosis, and ketosis can be avoided by adding lasalocid (LAS) to goats' feed (5).

Lasalocid is an effective polyether ionosphere antibiotic, widely used as a feed additive in the livestock industry to enhance weight gain and feed consumption efficiency, by reducing the amount of feed intake without affecting the daily weight gain of ruminants (6, 7). Its chemical structure consists of a cyclic polyether ring system with a carboxylic acid group and a piperidine ring attached. The properties of lasalocid include its ability to selectively bind and transport metal cations, particularly sodium, and potassium across cell membranes. This disrupts the ion balance in the target microorganisms, leading to their death. Lasalocid is also highly hydrophobic and lipophilic, easily penetrating cell membranes and accumulating in target cells. It has low toxicity to mammals, making it safe for use in animal feed. However, it is toxic to certain bird species and should not be fed to them.

Lasalocid increases the efficiency of energy utilization and minimizes the energy cost by inhibiting the breakdown and deamination of ruminal amino acid, increasing propionate volatile fatty acid (VFA), and decreasing acetate VFA, ammonia, and methane (8–11). Lasalocid can also modulate acid/base status by translocation of ions across cellular membranes post absorption (12). Lasalocid can also alter metabolic activity, growth performance, nutrient digestibility, rumen characteristics, blood metabolism, and enzyme activity including aspartate aminotransferase (AST) (13–15). Lasalocid has been shown to have positive effects on lipid metabolism in cows and sheep, including an increase in high-density lipoprotein (HDL) levels. Higher levels of HDL have been associated with a lower risk of cardiovascular disease, indicating the potential benefits of lasalocid supplementation in improving animal health and productivity (16). Recent literature reviews have highlighted the potential benefits of LAS supplementation for improving animal performance and welfare. For example, a review by Marques and Cooke (17) found that LAS, including lasalocid, can improve feed efficiency and reduce methane emissions in ruminants. A review also suggested that LAS can improve nutrient utilization in ruminants. Li et al. (18) and Hristov et al. (19) found that LAS can improve feed efficiency and nutrient utilization, and reduce methane emissions in ruminants. In sheep, Lippy et al. (20) indicated that dietary inclusion of LAS with zeranol improved overall BW, ADG, feed efficiency, and meat characteristics such as hot carcass weight and loin eye area in hair sheep. Polizel et al. (21) confirmed that the inclusion of 8 mg kg^−1^ of dry matter diet (DM) of monensin improves ruminal fermentation and plasma parameters, resulting in greater growth performance in lambs. Lasalocid has no harmful impact on lamb welfare (22). However, little information is available on the effects of LAS supplementation on goats. The effects of LAS on growth performance will depend on its level in animal feed. To the best of our knowledge, there is only one published research that tested only one level of lasalocid (30 mg/head/day) in growing dairy goats fed a high-forage, low-protein diet (12). Therefore, current research is the first experiment to evaluate the effects of increasing levels of lasalocid supplementation on growth performance, serum biochemistry, ruminal fermentation profile, *in vitro* digestibility, and gas production of growing goats. We hypothesize that lasalocid supplementation at different levels may have positive, negative, or no effect on growth performance, serum biochemistry, and *in vitro* ruminal fermentation profile of growing goats. Therefore, the aim of this study was to investigate the effect of increasing levels of LAS supplementation on growth performance, serum biochemistry, ruminal fermentation profile, *in vitro* digestibility, and gas production of growing goats.

## Materials and methods

### Animal Welfare and ethics clearance

The study was undertaken at King Saud University, Riyadh, Saudi Arabia. The use of animals and procedures adopted in this study was in accordance with the Animal Welfare Act of Practice for the Care and Use of Animals for Scientific Purposes and approved by the Research Ethics Committee, King Saud University (REC-KSU; Ethics Reference No: KSU-SE-21-82).

### Animals and management practices

A total of 60 Aardi male goats (mean BW 17.12 ± 0.51 kg; 3 months old) were used in an 84-day trial. In total, 14 days before the study commenced, animals were purchased from the local livestock market and then transported to the Experimental Station of the Animal Production Department, College of Food and Agriculture Sciences, King Saud University, Riyadh. On the day of arrival, goats were immediately weighed, ear-tagged, vaccinated against endemic diseases (One Shot Ultra™, Zoites, USA), and treated for internal and external parasites (Ivomec Super Injection, Merial, USA). Thereafter, animals were randomly divided into 20 replicates (five replicates/treatment) of 3 goats/replicate, and housed for group feeding in shaded pens. All goats were offered the same diet (the basal diet; Table 1), which was formulated to meet the nutritional requirements [NRC, 2007 (23)] of growing goats (2.5% of the initial body weight throughout the 84-day feeding period) two times daily at 0800 and 1,500, and water was provided *ad libitum*. At the beginning of the experiment (day 1), the goats were randomly assigned to one of four dietary treatments (15 goats/treatment), which were as follows: (1) the basal diet without LAS supplementation; LAS0); (2) the basal diet supplemented with 10 ppm LAS/kg DM (LAS10); (3) the basal diet supplemented with 20 ppm LAS/kg DM (LAS20), and (4) the basal diet supplemented with 30 ppm LAS/kg DM (LAS30). The LAS as lasalocid sodium was provided by Avatec^®^ 150G (Zoetis, Parsipanny, NJ, USA). The calculated amounts of LAS for each group were first mixed with other ingredients such as vitamins and mineral salts, and mixed very well for a period of time then thoroughly mixed with small quantities of basal diet. Then, the basal diet increased gradually with good and careful mixing until reached the final amount to be sure that every few grams of basal diet had the required amount of LAS. Finally, the pelleting process was done and the feed was presented as a complete pelleted diet.

**Table 1:** Ingredients and chemical composition of the basal diet used in the experiment.

**Item**	**Content**
**Ingredients, % of dietary dry matter**	
Corn, grain	30.70
Alfalfa hay	23.00
Soybean meal	11.65
Wheat bran	18.00
Wheat straw	7.00
Salt	0.83
Dicalcium phosphate	1.72
Acid buffer	0.80
Limestone	3.00
Molasses	3.00
Mineral & Vitamin Premix2	0.30
**Nutrient composition, dry matter basis**	
Dry matter, (%)	90.33
Ash, %	7.27
Crude protein, %	17.00
Ether extract, %	1.81
Neutral detergent fiber, %	27.44
Acid detergent fiber, %	15.40
Metabolizable energy, MJ/kg	8.03

### Feed analyses

Feed from each treatment was sampled before the study and monthly during the study, and samples were frozen at −5°C. At the end of the study, feed samples were pooled (5%) and analyzed for nutrient composition at King Saud University Laboratories. Dry matter (DM) content was determined by drying samples in an oven at 100°C for 24 h, while ash content was determined by incinerating samples at 550°C for 3 h in a muffle furnace AOAC (24). Crude protein (CP) was measured using an elemental analyzer. Neutral detergent fiber (NDF) and acid detergent fiber (ADF) were determined according to methods described by Van Soest et al. (25) and the AOAC (26) (method no. 973.18 C, 1990), respectively.

### Growth rate and feed efficiency

The weights of the offered feed and feed refusals were measured weekly, and then feed intake was calculated on a DM basis. Goats were weighed using an electronic small-animal scale for small ruminants, before morning feeding, at 07:30 h on day 1 of the study, and every 2 weeks thereafter until the end of the study. The average daily gain (ADG) was calculated for each lamb. The feed conversion ratio for each animal was calculated and expressed as DMI-to-BW ratios.

### Blood sample processing and analysis

Blood samples were collected from all goats (*n* = 15/group) in the morning before the feeding *via* jugular venipuncture on days 1, 42, and 84. At each collection, 10 mL aliquots of blood were taken into the vacutainer tubes without additives for serum collection. Serum was obtained by centrifugation at 2,400× g for 15 min at 4°C and then frozen at −20°C until further analysis. Serum concentrations of glucose, total protein, albumin, globulin, urea, creatinine, total cholesterol, high-density lipoprotein (HDL), low-density lipoprotein (LDL), aspartate aminotransferase (AST), alanine aminotransferase (ALT), and creatine kinase (CK) were analyzed using commercial kits (Randox Laboratories, Antrim, United Kingdom) and a microplate reader (Multiskan EX, Thermo Fisher Scientific Inc., Waltham, MA, USA) according to the manufacturer's instructions.

### Rumen fermentation characteristics

At the end of the experiment, ~50 mL of rumen fluid samples were collected from each goat (*n* = 15/group) by using an oral stomach tube fitted to a vacuum pump designed for the purpose. Samples were collected 1 h before the morning feeding. The rumen fluid was squeezed through four layers of cheesecloth, transferred into pre-warmed thermoses and immediately transported to the laboratory, and the pH of the rumen fluid was immediately measured using a digital pH meter (Hanna Instruments Inc., Woonsocket, RI, USA). The volatile fatty acids (VFA) and ammonia-N (NH3-N) were determined in the culture media using a sterilized jar. In total, 5 mL of sample from each jar was taken, placed directly in an ice bath, and frozen at −20°C prior to analysis. Then, samples were mixed with 1 mL of 25% meta-phosphoric acid and centrifuged for 20 min at 3,000 rpm at 4°C to produce a clear supernatant. After that, 1 ml of the collected supernatant was filtered through a 0.2 μm PTFE syringe filter (Acrodisc CR 25 mm Syringe Filter, PALL Life Sciences), and moved to a 1.5 mL glass chromatography vial (Agilent 1100 Series HPLC system). The volatile fatty acids (VFAs) composition (acetic acid, propionic acid, butyric acid, iso-butyric acid, valeric acid, and iso-valeric acid) was analyzed by gas chromatography (Shimadzu Scientific Instruments Inc., Columbia, MD), and the 2-ethylbutyric acid solution was used as an internal standard (27). The volatile fatty acids were performed as described by Cui et al. (28), and were represented as mM/1 mL samples. A sample was taken from each jar and centrifuged at 10,000 RPM for 15 min at 4°C. The ammonia N concentration of NH3-N was determined after being acidified with 0.5 mL of 0.1 N HCl using a spectrophotometer (Perkin Elmer, Waltham, MA, USA).

### *In vitro* gas production and digestibility of dry and organic matters

*In vitro* gas production was carried out according to Tedeschi et al. (29). The diluting buffered mineral solution was prepared and placed into a water bath at 39°C under continuous flushing with CO_2_. Dietary samples (200 mg) from each treatment (*n* = 15/treatment) were accurately weighed and put into calibrated plastic syringes. The collected rumen fluids (*n* = 15/treatment) were homogenized and again strained through four layers of cheesecloth and kept at 39°C in a water bath with continuous CO_2_ pumping. The rumen fluid together with the diluting buffered mineral solution was mixed in a ratio of 1:2. The mixture was kept and stirred under CO_2_ in a water bath at 39°C. Then 30 mL of the buffered rumen fluid was dispensed into each syringe containing the weighed diet samples, fitted with a plunger, and immediately placed in a rotor inside the incubator. A total of three syringes containing only 30 mL of the buffered rumen fluid served as blanks. Amounts of gas production were recorded at 2, 4, 6, 8, 10, 12, 14, 16, 18, 20, 22, and 24 h of incubation in a water bath (Thermo Fisher Scientific, model 2873, Waltham, MA, USA) at 39°C. During this process, the syringes were shaken for 30 s every 2 h. The amount of gas production was read from the calibrated scale on the plastic syringes. The actual total gas production was calculated by subtracting the gas produced in blank syringes from the total gas produced in syringes containing feed substrates and buffered inoculum.

### Statistical data analysis

All goats were randomly divided into experimental units for dietary treatments (LAS0, LAS10, LAS20, and LAS30) using a completely randomized design. The data of this trial were analyzed using one-way ANOVA with general linear model procedures (GLM) of SAS 9.4 software (30), as a completely randomized design. Additionally, orthogonal polynomial contrasts were used to determine the linear and quadratic effects of treatments on the evaluated parameters. The Duncan multiple range test was used to compare the means of the dietary treatments when the effect of the dietary treatment was considered statistically significant at *P* < 0.05.

## Results

The growth performance of growing goats fed dietary treatments with lasalocid (LAS) is presented in Table 2. The overall body weight gain (BWG) and average daily gain (ADG) were significantly higher in the LAS30 group (*P* < 0.04) than that in the other dietary-treated groups. Linear and quadratic effects of LAS were non-significant for all growth performance parameters (Table 2).

**Table 2:** Effects of different levels of lasalocid supplementation on growth performance of growing goats (*n* = 15/group).

**Variables, unit**	**Dietary treatments**				**SEM**	***P*** **-value**		
	**LAS0**	**LAS10**	**LAS20**	**LAS30**		**ANOVA**	**Linear**	**Quadratic**
Initial BW, kg	17.29	16.93	17.25	17.08	0.51	0.96	0.97	0.63
Final BW, kg	35.42	34.31	36.72	37.54	1.27	0.28	0.49	0.25
BW gain, kg	18.12^a^^b^	17.36^b^	19.50^a^^b^	20.45^a^	0.99	0.04	0.32	0.19
ADG, g/d	211^a^^b^	201^b^	226^a^^b^	237^a^	8.13	0.04	0.32	0.19
DMI, g/d	744	725	733	732	19.81	0.48	0.44	0.19
FCR	3.64	3.68	3.38	3.18	0.21	0.23	0.38	0.48

a, b Within a row, means without a common superscript differ (*P* < 0.05).

LAS, the basal diet without LAS supplementation; LAS10, the basal diet supplemented LAS at a level of 10 ppm; LAS20, the basal diet supplemented LAS at a level of 20 ppm; LAS30, the basal diet supplemented LAS at a level of 30 ppm; BW, Body weight; ADG, Average daily gain; DMI, Dry matter intake; FCR, Feed conversion ratio; SEM, Standard error of means.

**Figure 1:**
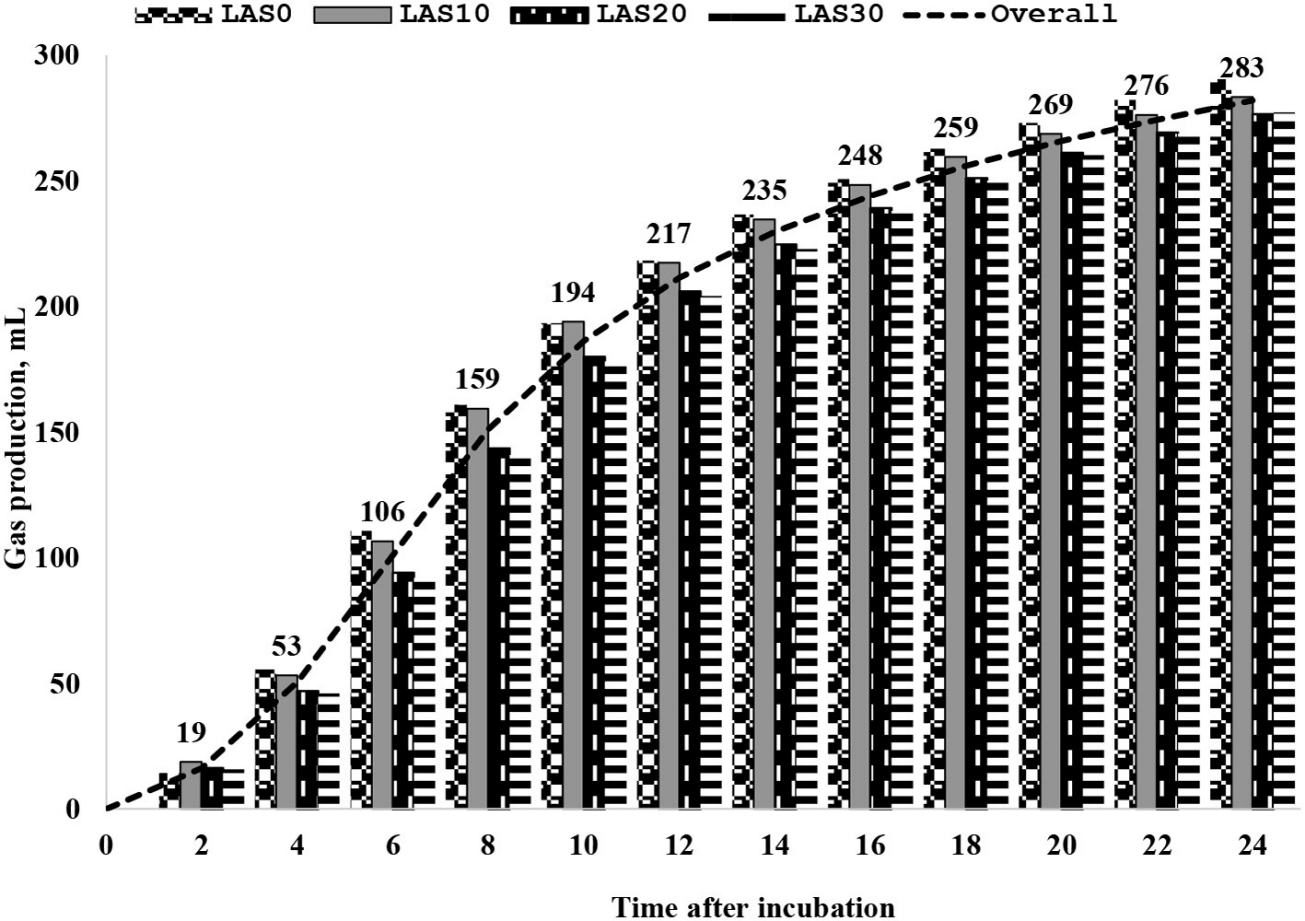
Cumulative total gas production every 2 h after incubation *in vitro* digestion for basal diet contained different levels of lasalocid. LAS, the basal diet without LAS supplementation; LAS10, the basal diet supplemented LAS at a level of 10 ppm; LAS20, the basal diet supplemented LAS at a level of 20 ppm; LAS30, the basal diet supplemented LAS at a level of 30 ppm.

The effect of LAS supplementation on serum biochemistry and enzymatic activity of growing goats is presented in Table 3. The serum concentrations of high-density lipoprotein (HDL) were significantly (*p* < 0.05) higher in the LAS20 group than in other groups with linear and quadratic effects, while low-density lipoprotein (LDL) concentration was significantly (*p* < 0.05) lower in the LAS20 group than in other groups LAS0 and LAS30 with a linear effect. The ratio between LDL and HDL in serum decreased (*P* < 0.05) in goats fed LAS20 compared to the other treated groups with linear and quadratic effects. The results show no significant effects of supplemental LAS on the glucose, total protein, albumin, globulin urea, creatinine, alanine aminotransferase (ALT), alkaline phosphatase (ALP), creatine kinase (CK), total cholesterol, and triglyceride levels. Significant effects were observed for lipoproteins, including high-density lipoprotein (HDL), low-density lipoprotein (LDL), and the ratio between HDL and LDL. The results also showed that aspartate aminotransferase (AST) concentration was higher (*P* < 0.05) in the diet of LAS 10 than that of LAS20 and control (LAS0). Different levels of lasalocid supplementation had no effect on the ruminal fermentation profile and *in vitro* gas production and nutrient digestibility (Table 4 and Figure 1).

**Table 3:** Effects of different levels of lasalocid supplementation on serum concentrations of metabolic and enzymatic variables of growing goats (*n* = 15/group).

**Variables, unit**	**Dietary treatments**				**SEM**	***P*** **-value**		
	**LAS0**	**LAS10**	**LAS20**	**LAS30**		**ANOVA**	**Linear**	**Quadratic**
Glucose, mg/dl	67.16	70.03	68.7	68.76	1.79	0.77	0.59	0.36
Total protein, g/dl	3.95	4.22	4.09	4.2	0.14	0.48	0.49	0.21
Albumin, g/dl	2.11	2.12	1.99	2.14	0.06	0.37	0.22	0.33
Globulin, g/dl	1.84	2.11	2.09	2.06	0.11	0.27	0.11	0.29
Urea, mg/dl	20.33	23.17	20.74	21.37	0.88	0.08	0.74	0.01
Creatinine, mg/dl	0.72	0.81	0.78	0.81	0.05	0.58	0.48	0.28
Total cholesterol, mg/dl	87.77	85.2	89.29	90.18	3.08	0.63	0.73	0.34
HDL, mg/dl	41.05^b^	41.39^b^	50.29^b^	41.77^b^	1.29	0.001	< 0.0001	0.01
LDL, mg/dl	34.81^b^	30.83^a^^b^	25.65^b^	35.95^b^	2.49	0.04	0.02	0.85
LDL: HDL	0.92^b^	0.78^b^	0.54^b^	0.90^b^	0.09	0.01	0.01	0.05
Triglyceride, mg/dl	59.52	64.9	66.77	62.32	2.66	0.24	0.05	0.56
AST, U/L	65.23^b^	71.64^b^	65.94^b^	70.54^a^^b^	1.86	0.05	0.81	0.01
ALT, U/L	19.58	20.87	21.13	21.28	0.97	0.59	0.26	0.63
ALP, U/L	230.1	260.5	211.6	228.8	26.9	0.67	0.67	0.25
CK, U/L	0.75	0.73	0.56	0.64	0.08	0.26	0.08	0.38

a, b Within a row, means without a common superscript differ (*P* < 0.05). LAS, the basal diet without LAS supplementation; LAS10, the basal diet supplemented LAS at a level of 10 ppm; LAS20, the basal diet supplemented LAS at a level of 20 ppm; LAS30, the basal diet supplemented LAS at a level of 30 ppm; Alkaline phosphatase; AST, aspartate aminotransferase; CK, creatine kinase; HDL, high-density lipoprotein; LDL, low-density lipoprotein; SEM, Standard error of means.

**Table 4:** Effects of different levels of lasalocid supplementation on ruminal fermentation profile and *in vitro* gas production and nutrient digestibility (*n* = 15/group).

**Variables, unit**	**Dietary treatments**				**SEM**	***P*** **-value**		
	**LAS0**	**LAS10**	**LAS20**	**LAS30**		**ANOVA**	**Linear**	**Quadratic**
*In vitro* DM digestibility, %	72.81	78.24	75.44	77.05	2.18	0.38	0.42	0.16
*In vitro* OM digestibility, %	72.74	78.20	75.05	76.84	2.08	0.35	0.46	0.13
*In vitro* total gas production, mL	290.43	283.31	276.67	277.28	0.93	1.61	0.06	0.44
Ruminal pH	6.12	6.31	6.31	6.25	0.06	0.15	0.06	0.23
Total VFA, mM	52.07	54.51	54.43	52.21	2.14	0.76	0.46	0.64
**VFA composition, mM**								
Acetate, C_2_	29.19	30.30	29.96	29.17	1.41	0.92	0.71	0.68
Propionate, C_3_	11.49	12.31	12.32	11.64	0.43	0.42	0.21	0.46
Butyrate, C_4_	9.12	9.27	9.52	8.93	0.78	0.95	0.73	0.96
Iso-Butyrate	0.46	0.54	0.53	0.49	0.03	0.21	0.09	0.24
Iso-Valerate	0.90	1.00	1.00	0.95	0.08	0.36	0.12	0.43
Valerate	0.92	1.08	1.10	1.03	0.07	0.67	0.31	0.55
C_2_: C_3_ ratio	2.54	2.46	2.44	2.50	0.09	0.88	0.49	0.85
Ammonia-N, mM	6.74	7.79	6.75	7.38	0.81	0.75	0.99	0.32

LAS, the basal diet without LAS supplementation; LAS10, the basal diet supplemented LAS at a level of 10 ppm; LAS20, the basal diet supplemented LAS at a level of 20 ppm; LAS30, the basal diet supplemented LAS at a level of 30 ppm; DM, Dry matter; OM, Organic matter; VFA, volatile fatty acids; SEM, Standard error of means.

## Discussion

Numerous studies have shown that lasalocid (LAS) supplementation in feed can enhance feed consumption, weight gain, and feed efficiency in small ruminants (31–34), dairy heifers (35), and young calves (36). In the current investigation, the supplementation of 30 ppm of LAS to the diet of growing goats improved performance, as evidenced by an 11.71% increase in body weight (BW) gain and a 9.54% increase in average daily gain (ADG), without significant differences in initial and final BW or dry matter intake (DMI) compared to the control group. These findings support many of the findings that LAS supplementation led to an increase in BWG, ADG, DMI, and FCR in growing goats (12, 37). Additionally, a study conducted by Polizel et al. (38) indicated that LAS supplementation at 13 ppm led to increasing ADG in sheep and growing lambs. Therefore, our results suggest that LAS supplementation can improve feed efficiency (35) and increase BWG and ADG, which is consistent with previous studies (31, 39). However, it is uncertain whether the enhancement in performance observed resulted from an increased feed intake efficiency. As some reports have indicated that feeding LAS has no effect on the feed intake of goats (12, 40). In addition, LAS can improve animal performance by enhancing feed digestibility and nutrient utilization, modulating rumen fermentation, and reducing subclinical infections. For example, Crane et al. (33) found that LAS supplementation in the diet of growing lambs improved nutrient digestibility, growth, and carcass traits. Bhatt et al. (41) reported that LAS and monensin improved growth performance, nutrient digestibility, blood metabolites, and meat quality in Malpura lambs. Nonetheless, our findings suggest that LAS supplementation can be a useful tool for improving the growth and performance of goats, particularly at the level of 30 ppm in the diet.

The effects of lasalocid (LAS) supplementation on metabolic and enzymatic variables were investigated in goats; however, there were no significant changes in the levels of glucose, total proteins, albumin, globulin, urea, and creatinine among different groups, while there were some noticeable changes in other variables. It is possible that more time is necessary for seeing significant differences in lipoproteins profiles and enzymatic activity may have been noticed after LAS supplementation. Our results indicated that serum LDL was lower when goats were supplemented with LAS than in other treated groups. In contrast, goats fed LAS have a higher HDL, especially with the LAS20 group than the control goats. It means that LAS may be led to enhance energy metabolism and status and reduce energy demand, which is clear because no effects between groups for glucose and FVAs. Our results have the same trend as indicating that LAS may enhance energy metabolism and status and reduce energy demand. The results are consistent with previous studies (35), but contrary to a study conducted by Rajaian et al. (42) in sheep. In addition, a recent study demonstrated a significant increase in AST in goats treated with LAS (LAS10 and LAS30, respectively), it may be because supplementation improved energy utilization and liver functions as AST is known as a vital biomarker for liver health (9). These findings suggest that more time may be necessary to see significant differences in lipoprotein profiles and enzymatic activity after LAS supplementation. Overall, LAS appears to have positive effects on lipid metabolism and liver function in goats.

The *in vitro* digestibility of dry matter (DM) and organic matter (OM) did not significantly affect the levels of lasalocid (LAS) (10 to 30 ppm) in the rumen of goats fed experimental diets compared to those fed traditional diets (LAS0). These results differ from previous reports by Crane et al. (33, 34), who found that DM and OM digestibility improved in treated groups. However, no significant differences in volatile fatty acid (VFAs) composition between goats in the study and control groups, which may be due to the need for glucose as an energy source. However, dietary treatment with LAS was not significantly affected ruminal pH, total gas production, and ammonia-N between goats in treated groups and control. Our results had the same trend as the finding by Ribeiro et al. (43) that was found on sheep. In general, cumulative gas production *in vitro* digestion and fermentation trial trended to increase in goats fed different levels of LAS (LAS30) within 2 to 24 h periods, and concentrations of total gas decreased slightly and the last hour. Changes in gas production could be indicative of altered metabolic activity. The lower gas production observed in LAS-fed goats may indicate that LAS is an effective means of controlling acidosis in these animals. On the other hand, a tendency of lower total gas production in goats fed LAS could involve reducing metabolic acidosis. The observed variations in gas production are likely reflective of the goats' responses to LAS feeding and may suggest that LAS has an effect on the acid/base balance in these animals (12, 44).

## Conclusion

The results from the current study conclude that the addition of LAS (20–30 ppm/kg DM) to the goat's diet can improve the growth performance and lipoprotein profile. The supplementation of lasalocid with 30 ppm/kg DM improved growth performance by enhancing body weight gain. The addition of lasalocid with 20 ppm DM has a positive effect on lipoprotein profile (HDL and LDL) and enzymatic activity (AST) in growing goats. Different levels of lasalocid supplementation had no effect on the ruminal fermentation profile, *in vitro* gas production, and nutrient digestibility.

## Data availability statement

The raw data supporting the conclusions of this article will be made available by the authors, without undue reservation.

## Ethics statement

The animal study was reviewed and approved by the study was undertaken at King Saud University, Riyadh, Saudi Arabia, and the use of animals and procedures adopted in this study were in accordance with the Animal Welfare Act of Practice for the Care and Use of Animals for Scientific Purposes and approved by the Research Ethics Committee, King Saud University (REC-KSU; Ethics Reference No: KSU-SE-21-82).

## Author contributions

SB, AS, and MA-B: methodology, formal analysis, and writing—original draft. MAA and GS: conceptualization, methodology, and data curation. MMA, AE-W, FA-H, AS, and IA: investigation, review and editing, and as well as project administration. All authors have read and agreed to the published version of the manuscript.
